# Rotor angle stability enhancement using DDPG reinforcement agent with Gorilla troops optimized input scaling factors

**DOI:** 10.1038/s41598-025-91699-1

**Published:** 2025-03-23

**Authors:** Ahmed H. Yakout, Ahmed E. B. Abu-Elanien, Hany M. Hasanien

**Affiliations:** 1 Electrical Power and Machines Department, Faculty of Engineering, Ain Shams University, Cairo, 11517 Egypt; 2 Electrical Engineering Department, Faculty of Engineering, Alexandria University, Alexandria, 21544 Egypt; 3 Faculty of Engineering and Technology, Future University in Egypt, Cairo, 11835 Egypt

**Keywords:** Interarea oscillations, Local rotor angle oscillations, PSS, Rotor angle stability, Reinforcement learning, Electrical and electronic engineering, Energy grids and networks

## Abstract

This paper introduces a Reinforcement Learning (RL)-based Power System Stabilizer (PSS) with a Deep Deterministic Policy Gradient (DDPG) algorithm for rotor angle stability. The proposed stabilizer uses scaled values of the generator’s accelerating power, a derivative of accelerating power, integration of accelerating power, and generator real power as inputs. The stabilizer uses the DDPG algorithm to train The RL agent. Moreover, to further enhance the PSS performance, the scaling factors of the input observations are optimized using the Gorilla Troops Optimization (GTO) algorithm, which is known for its robustness, fast convergence. Furthermore, the RL reward considered is a discrete function that rewards the generators’ accelerating power samples when they are below a defined value. The proposed PSS is tested on three popular case studies: a Single Machine connected to an Infinite Bus (SMIB), Kundur’s four-machine system, and the IEEE 39 bus ten machine system. The proposed stabilizer performance is compared with three common IEEE common PSSs: the Multiband dw speed-based PSS (MB-PSS), the lead-lag dw speed-based PSS (dw-PSS), and the lead-lag dPa accelerating power-based PSS (dPa-PSS). MATLAB simulations prove that the proposed PSS performs better than the other PSSs. It shows higher transient stability capability than the compared PSS even with long duration faults.

## Introduction

Rotor angle stability is assumed to be one of the most essential stability classifications. It represents the ability of a synchronous generator to stay in synchronism with a power system after a small or a large disturbance. Instability is manifested by an unstable increase in the generator rotor angle and speed in oscillatory or aperiodic forms. A study of the rotor angle stability for small disturbances is called small signal stability^1,2^, while large disturbances, such as faults, are called transient stability^3^. When a disturbance is very close to a generator, the generator starts to oscillate locally against the whole system; hence, this type of oscillation is called local power system oscillations since oscillations. However, in case of a more significant fault in the middle of a network, some generators might oscillate against other distant generators; therefore, this phenomenon is called interarea oscillations. Depending on the fault’s location and clearance time, two or more areas might oscillate against each other, leading to the growth of power oscillations in transmission tie-lines. These oscillations may lead to losing system stability. Basics of multi-area stability can be found in^4–6^. Current research methods for improving rotor stability can be divided into three main groups. The first group includes adding Flexible AC Transmission System (FACTS) devices to the power system for damping rotor oscillations. The second is improving the generator controller performance. The third implements new types of Power System Stabilizers (PSSs) as an additional control that uses the change of machine speed and/or accelerating power to alter the machine excitation reference value within allowed limits (usually ± 0.15 p.u).

Concerning the usage of FACTS devices, a Static VAR Compensator (SVC) with a Proportional-Integral (PI) controller is suggested in^7^, and with a fractional order Proportional-Integral-Derivative (PID) controller in^8^ for system stability support. The SVC and fuzzy controllers are introduced in^9^ to enhance transient stability and damp harmful oscillations. STATCOM is coordinated with a PSS in^10^, and with a fuzzy based PSS in^11^ to improve the transient rotor stability. Moreover, the rotor stability is enhanced through a coordinated action between PSS and thyristor-controlled series compensator in^12^, and static synchronous series compensator in^13^. Coordinated control of SVC and static synchronous series compensator is presented in^14^ to enhance system stability. Reinforcement Learning (RL) agent was used to control a Thyristor-Controlled Series Capacitor (TCSC) for damping power system oscillations in a 4-machines system^15^. However, adding FACTS devices is considered an expensive solution compared with stabilizers and regular controllers.

The second research direction focuses on introducing new controllers or improving the existing controllers of the excitation system to enhance the system’s stability. The PID excitation controller is optimized by an ant colony algorithm in^16^, by Whale optimization in^17^, and by an improved version of the whale optimization algorithm in^18^. These optimized controllers showed better performance than regular PID controllers. Simulink optimization is proposed in^19^ to establish a PID controller for Automatic Voltage Regulator (AVR). The optimizer performed better than the local unimodal sampling algorithm, genetic algorithm, and water cycle algorithm. A modified African vulture-optimized PI controller for AVR applications is proposed in^20^. Four types of PID controllers (ideal, real, fractional-order, and second-order derivative controllers) are optimized using manta ray foraging algorithm combined with simulated annealing in^21^. Results showed the enhanced transient performance of the system. In^22^, the application of a Jaya optimized Fractional-Order Proportional Integral Derivative (FOPID) controller in the automatic voltage regulator system is explored. Results showed an enhancement in the system dynamic stability. Davut et al. demonstrated that the artificial rabbit optimizer-based FOPID controller with a double derivative controller outperform conventional controllers^23^. An optimized fractional adaptive controller is proposed in^24^. This controller exhibited low rising and settling times with a smooth capability to track dynamic reference changes. An artificial neural network-based AVR system is proposed for an isolated generator feeding a group of loads^25^, whereas a multilayer feedforward neural network is used to improve the AVR performance of a single machine connected to an infinite bus (SMIB)^26^. However, the aforementioned algorithms are not tested for multi-machine large interconnected systems. Local and interarea rotor angle oscillations are damped using a marine predator optimized cascaded PID controller^27^. Results demonstrated low overshoot fast-tracking capability of reference values. However, perfect tuning of PI and PID controllers needs extensive experience. Moreover, even with optimized PI and PID controllers, their robustness depends on the specified operating conditions.

PSSs are used for rotor angle stability due to their relatively low cost and uncomplicated implementation^5^. Adding PSS enhances the damping characteristics of generator during disturbances^28^. Popular PSSs are the Multiband and Lead-Lag structures^6^. Many articles are presented to design PSSs. PSS parameters are tuned by Jaya algorithm in^29^, by a modified genetic algorithm^30^, and by Ziglar Nicolas method in^31^. However, the PSS performance is tested on SMIB only. Moreover, the PSS performance is not compared with other PSSs. Particle swarm and firefly algorithms^32^, and mayfly algorithm^33^ are used to optimized PSS parameters. Nevertheless, results are not compared with other PSS types in^32^, and not tested for large disturbances in^33^.

Deep learning is not extensively utilized in the literature for rotor frequency control. The current deep learning approaches are focused on tuning parameters of conventional PSS. Convolutional neural network is used to tune fractional order PID-based PSS control in^34^. Deep neural network is used to tune parameters of multi band PSS in^35^. However, these methods are tested on SMIB only. Convolutional neural network and RL are used to tune parameters of PSS in^36,37^. However, Convolutional neural network and RL agent are not used as the main PSS. It was used to provide the best parameters for the conventional PSS. Therefore, its performance cannot bridge the gap between conventional PSS and the self-learning, self-adaptive capabilities of deep learning agent-based PSS. When the PSS itself is an RL agent, it can use the self-learning and adaptability properties of the RL agent to stabilize different system configurations with wide range of fault types. Comparative studies of various power system stabilizers are summarized in^38,39^. The study showed the immature usage of deep learning methods in PSSs. Table 1 presents a summary of the rotor angle stability approaches.

**Table 1:** Summary rotor angle stability approaches.

	Rotor angle control approach		
	Controllers with FACTS devices	Enhancement of existing controllers	Using power system stabilizers (PSSs)
Idea of operation	FACTS devices are added to assist rotor to regain stability after disturbances.	Development of advanced controllers, improvement of existing controllers, and combining different controllers to keep system stability.	Using PSSs as additional controller to ensure machine stability.
References	^7–15^	^16–27,40,41^	^28–39^
Pros	Facts devices help machines to restabilize quickly after large disturbances.	Modern controllers provide acceptable stability options for different machine types.	They provide an inexpensive and uncomplicated stability solution for different machine types and system sizes.
Cons	Expensive FACTS devices increase system complexity and cost.	• Using highly complicated controllers and combining more than one controller increases the system operational complexity.	• Some PSSs do not perform well for different system sizes and disturbance types.
		• The performance of regular controllers may be unacceptable for large systems with problematic faults.	• Researchers did not take full advantage from deep learning approaches. Deep learning agent is not used as a PSS.

A new RL-based accelerating power (RL-dPa) with Deep Deterministic Policy Gradient (DDPG) algorithm PSS is introduced for rotor angle stability in this research. RL agent contains a deep neural network that maps between inputs (observations) and the targeted control output. Successful training leads to a large long-term RL reward (i.e., minimum change in accelerating power), and improving system stability. DDPG-RL-based agent exhibits stable operation, fast convergence, and effectiveness in solving related power system problems, especially frequency stability^42^ and voltage control^43^. The input observations of the RL-based PSS are multiplied by scaling factors to enhance the performance of the proposed PSS. Gorilla Troops Optimization (GTO) is employed to optimize the observations’ scaling factors of the RL-based PSS. A three different case studies are considered to test ability of the proposed PSS to work in different system sizes and adapt to changed configurations. The first case study is the SMIB power system, which tests the proposed PSS for local oscillations. The second is Kundur’s two-area four-machine system, which represents a medium-size case study of interarea oscillations. Finally, the IEEE-39 bus is studied to verify the ability of the proposed PSS to damp interarea oscillations in multi-generators large systems. Main features and contributions of this research work are itemized as follows:

1. RL agent with DDPG algorithm is proposed as the main PSS to deal with rotor angle stability problems.
2. The RL controller’s performance is enhanced by introducing optimized scaling factors for all inputs to the PSS. Optimization is done via GTO, which is a recent optimization technique that has good robustness and fast convergence characteristics.
3. The proposed PSS performance is compared with common PSSs including Multi-Band PSS, speed-based Lead PSS, and accelerating power-based Lead PSS. The performance results of the proposed controller were superior.
4. The proposed PSS exhibits notable results when applied to SMIB, Kundur’s two-area four-machine system, and the IEEE multi-machine 39-bus system, which proves its ability to work in different system scales.
5. The DDPG-RL controller shows outstanding learning, adaptation, and robustness characteristics under different power system sizes and configurations. The controller is trained for the SMIB case only; however, it provides outstanding performance for other test cases.

The remaining of this paper is structured as follows: section “DDPG- RL-based PSS with optimized observations” presents the proposed DDPG-RL based PSS, section “Test power systems” gives details about test systems, section “Simulation results” covers the simulation results of applying the proposed PSS to the test systems, and finally, section “Conclusion” concludes this paper.

## DDPG- RL-based PSS with optimized observations

This section introduces the design details of the RL-based PSS. It consists of two stages: stage one provides details of DDPG-RL agent, while stage two presents optimization of observations’ scaling factors using GTO.

### Stage 1: DDPG RL-based PSS

RL is a machine learning mechanism that uses an agent (controller in our case), constituting a learning method and a Policy. The learning method is used to train the policy to take the correct actions (controller output) based on observations (controller inputs) of the environment (controlled system). The learning methodology measures and tries to optimize a calculated reward (objective function) to obtain a good-performing agent. The RL agent training is based on the inputs from the environment, which is the power system under control. The agent’s (controller’s) actions are enhanced using the reward function. If the RL control actions are correct, the reward increases, while wrong actions penalize the agent by reducing the reward. DDPG and SAC (soft actor-critic) learning algorithms have an advantage over other learning algorithms, which is the ability to work correctly with continuously changing and large problems. DDPG is selected because it has faster convergence characteristics^44,45^; therefore, it is a suitable for load frequency control problems^46^. Moreover, the DDPG learning algorithm is not programmed for correct action, as it should find it without supervision. Nevertheless, humans interfere in adjusting the rewards and penalties to avoid unrealistic rewards. The DDPG algorithm can be considered in the middle between supervised and unsupervised machine learning methods. Therefore, it avoids supervised learning problems such as wrong outputs for untrained cases. Moreover, the issue of the wrong categorization of unsupervised learning is significantly reduced by adjusting the limits of the reward function. The proposed RL-based PSS uses deep neural networks as a policy (i.e., it trains a deep neural network to build function approximators). The learning algorithm updates the DDPG policy to map between the system’s observations (RL agent inputs) and the correct action, which would lead to a maximum reward.

During Disturbances like faults, imbalance occurs between the generator input mechanical power and the electrical power output. This imbalance is expressed by the accelerating power (ΔPa), which forces the system to accelerate and change its speed according to the system’s inertia. For system stability, accelerating power should be reduced to zero; hence, it is important to supply the RL-based PSS with (ΔPa) as an input. The integral of the accelerating $(\smallint \;\Delta {\text{Pa}} {\text{dt}})$ power allows the PSS to make phase compensation, while the derivative of the accelerating power (d(ΔPa)/dt) provides the PSS with rate of change of accelerating power. The derivative of the accelerating power, together with the accelerating power itself, indicates the severity of the disturbance. Therefore, $(\smallint \;\Delta {\text{Pa}} {\text{dt}})$ and (d(ΔPa)/dt) serve as the second and third inputs to the PSS. Moreover, electrical power (Pe) is used as the fourth input to ensure that the PSS restores it to same value before the fault.

The Reward function is a discrete function with a sampling frequency of 100 Hz and a calculation time span of 30 s. It is formulated as follows:

1 $${\text{Reward}} = {1}0 \, \left( {\left| { \, \Delta {\text{Pa }}} \right| < 0.0{\text{1 p}}.{\text{u}}.} \right) - \left( {\left| { \, \Delta {\text{Pa }}} \right| \ge 0.0{\text{1 p}}.{\text{u}}.} \right) - {1}00 \, \left( {\omega \le 0.{\text{96 p}}.{\text{u}}.{\text{ or }}\omega \ge {1}.0{\text{4 p}}.{\text{u}}.} \right)$$

The accelerating power should be always below ± 0.01 p.u. to ensure stable generator operation. Therefore, the accelerating power sample is rewarded by 10 points if its absolute value is lower than 0.01 p.u., and penalized by − 1 if higher to train the RL agent on removing accelerating power. Moreover, the reward is penalized by − 100, and the simulation stops if the generator’s speed deviates from its rated value by ± 4% which corresponds to decrease in the frequency below 48 Hz, or increase above 52 Hz in 50 Hz system. These frequency deviations are unacceptable and would lead to generator trip by under/over frequency relay. Accordingly, The DDPG learning stops due to wrong learning. Finally, the output of the PSS to the exciter is limited to ± 15% to prevent the generator voltage from exceeding its permissible limits. The data is sampled such that one sample is taken every 0.01 s. One episode of training comprises a 30 s simulation which represents 30/0.01 = 3000 samples per episode. If all accelerating power samples are lower than 1%, which means acceptable value of accelerating power, then every sample would be rewarded a 10. Hence, a total Reward of 3000 × 10 = 30,000 will be given in this episode. The Reinforcement learning process was adjusted to stop if the total reward reached a value above 29,000, as it is difficult to reach the 30,000 due to the large used disturbances (in most of our simulation a short circuit is applied, and then cleared by the disconnection of the faulty line). The training of the RL stopped at episode 125 when the reward function reached 29,362 (above 29,000 the stopping criteria). It took 1 h 52 min and 30 s on a personal computer, Intel -core i5, 10^th^ generation, 2.5 GHz, 16 GB RAM.

### Stage 2: optimization of the observations’ scaling factors using GTO

**Fig. 1:**
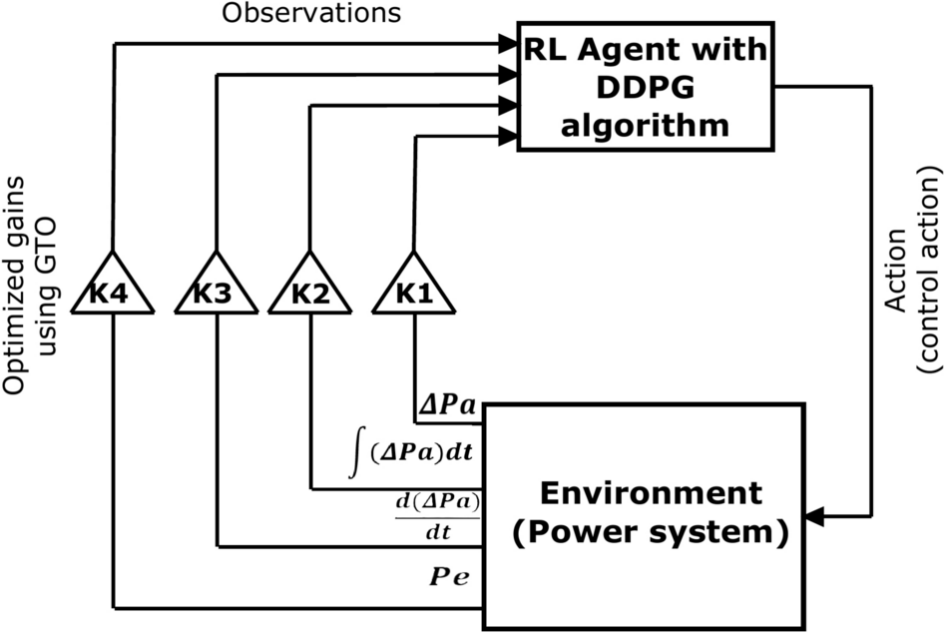
DDPG-RL PSS with scaled observations.

Since each input of the four inputs does not have the same importance with respect to the system stability, the observed values of ΔPa, ʃΔPa dt, d(ΔPa)/dt), and Pe are multiplied by four scaling factors (K1, K2, K3, K4). After extensive simulations, it was found that the scaling factors cannot exceed 20 for any system size; therefore, the scaling factor range is assumed between (0, 20). The scaling factors are optimized using GTO such that the inputs that have a larger effect on the control signal are given a higher scaling factor value and vice versa. This optimization process is performed as a second stage after training the DDPG -RL agent to boost the controller’s performance. Figure 1 depicts the operation of the proposed PSS. The trained RL agent with DDPG learning algorithm receives the four observations from the power system. The observations are multiplied with the optimized scaling factors. The DDPG-RL agent issues a control signal to the system based on the scaled observations.

GTO is a recent metaheuristic optimization approach inspired by the behavior of gorillas troops^47^. In this technique, the gorillas follow the group’s strongest male, known as the silverback (best solution). On the contrary, they move away from weaker gorillas, which represent non-optimal solutions. GTO was chosen in this study due to its robustness and quick convergence characteristics^47^, especially in stability problems^42^. Since it uses several options to update the gorillas’ positions, it has less probability to stuck in a local optimum. Moreover, it is a robust technique as it can solve high-dimensional optimization problems^47,48^. According to a study conducted^49^, GTO has faster convergence, lower mean square error, and lower standard deviation of error compared to various metaheuristic optimization techniques. Furthermore, as evidenced by the PSS results, the GTO optimized PSS gives excellent results as compared with other controllers. The GTO consists of an exploration phase and an exploitation phase. In the exploration phase, gorillas’ positions are updated based on one of the three situations. These situations are evaluated according to a random number “rn” between [0, 1], which determines the exploration alternative. If *rn* < p, gorillas move to a new unexplored place; if rn ≥ 0.5, gorillas move towards other gorillas to form a group; and if rand > 0.5, gorillas move towards a known area. The factor *p* represents the probability of moving to new places, and its value is 0.03, according to^47^.

2 $$CX\left( {t + 1} \right) = \left\{ {\begin{array}{*{20}c} {\left( {UL - LL} \right) r_{1} + LL if rn < p } \\ {\left( { r_{2} {-}D} \right)X_{r} \left( t \right) + S \times V if rn \ge 0.5 } \\ {X\left( t \right){-}S\left( { S \left( { X\left( t \right){-}CX_{r} \left( t \right)} \right) + r_{3} \left( { X\left( t \right){-}CX_{r} \left( t \right)} \right)} \right) if rn < 0.5} \\ \end{array} } \right.$$

where CX(t + 1) represents the vector of gorillas’ candidate positions in the next iteration, UL and LL are the top and bottom limits of the variables in solution space, respectively, X(t) is the present vector of positions at iteration t, ${\text{X}}_{\text{r}}$(t) is a randomly chosen number of gorillas from the current population, CX_r_(t) is an arbitrarily selected one gorilla’s position vector taken from the candidate solution at iteration t, r_1_, r_2_, r_3_ are random variables that take a value from 0 to 1, and S and V denote the movement of the silverback gorilla. The value of D signifies a scaling for exploration and a selector for the exploitation phase^47^.

3 $$D =\left(\text{cos}\left(2\times {r}_{4}\right)+1\right)\times \left(1 -\frac{t}{Mt}\right)$$

where t is the present iteration, Mt is the upper limit of iterations, and r_4_ is a random value between [0, 1]. The variable D decreases with the progress of iterations to narrow the solution space towards the best gorillas.

S and V can be found as follows:

4 $$S=\text{D}\times {\text{r}}_{5}$$

5 $$V=\text{N}\times \text{X}\left(\text{t}\right)$$

where, r_5_ and N are random values between [−1 to1] and [−D, D], respectively.

In the exploitation phase, either all gorillas follow Silverback commands to explore food locations, or male gorillas fight each other to take adult females’ attention. The first option occurs if D $\ge$ E, where E is a parameter that is equal to 0.8, according to^47^. Moreover, the equation representing this option is as follows xx^47^:

6 $$CX\left( {t + 1} \right) = S \times M \times \left( { X \left( t \right){-} X_{silverback} } \right) + X \left( t \right)$$

where ${X}_{silverback}$ denotes the positions of the silverbacks (best solution). M is a variable that relies on the candidate solution of iteration t and the number of gorillas in a solution vector.

**Fig. 2:**
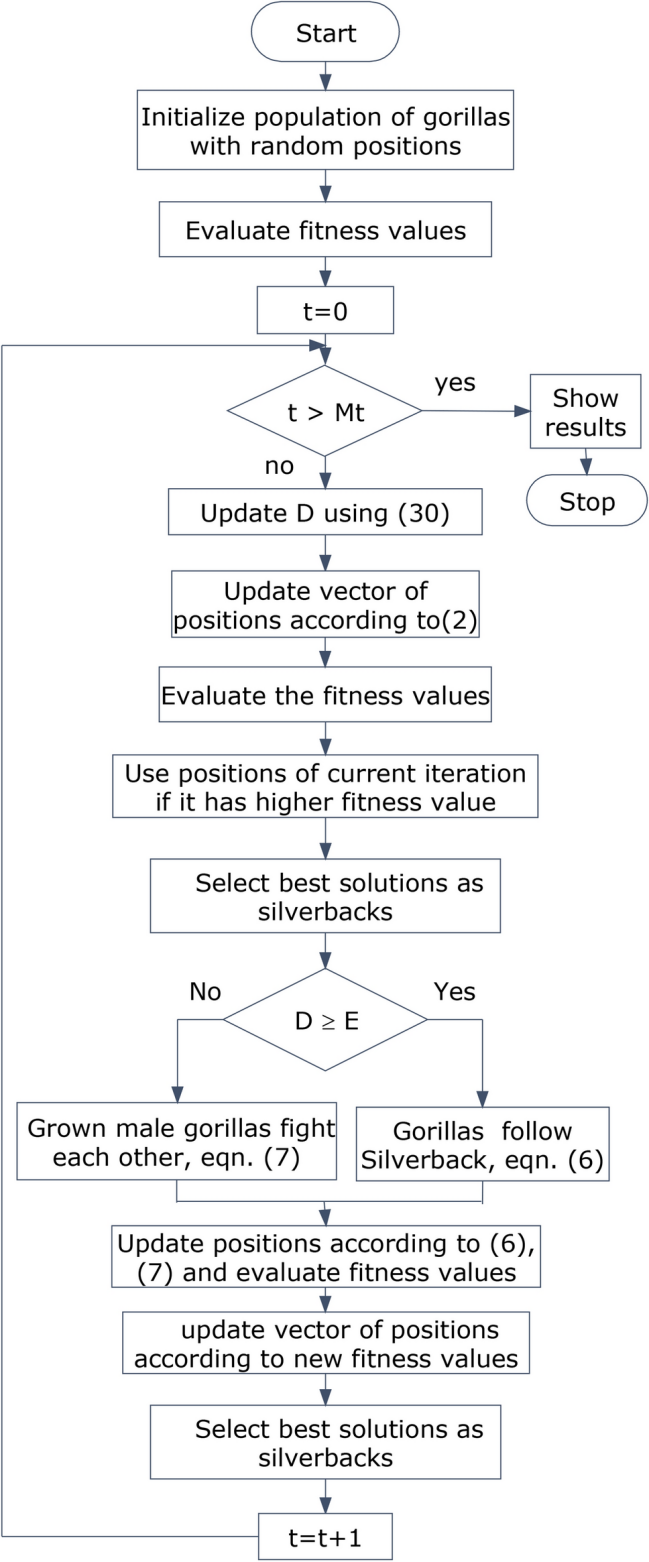
Gorilla troops optimization flowchart.

In the second option, adult male gorillas fight to win females. This option is selected if D < E. This option can be presented as follows^47,49^:

7 $$CX\left( i \right) = X_{silverback} {-} \left( { X_{silverback} \times R {-} X\left( t \right) \times R} \right) \times T$$

where $CX\left(i\right)$ is a candidate position for gorilla i in the troop, R is a factor that represents impact force, and T is a coefficient that represents violence level. At the end of exploitation, the fitness function is evaluated for X(t) and CX (t). If fitness value of CX (t) > X(t), CX (t) vector replaces X(t). Otherwise, nothing changes. Afterward, the exploration phase begins again for iteration (t + 1), and so on. This process is repeated until the last iteration (Mt). The optimal solution is the most updated value of X(t). Figure 2 is a flowchart demonstrating the GTO algorithm’s main steps.

The optimum scaling factors differ from system to another. The process of obtaining optimum scaling factors is carried out once for the system before applying the proposed PSS. Hence, it is applied offline; moreover, the optimum scaling factors do not change with the change of operating conditions, even under faulty conditions. Consequently, the optimization process neither interferes with the online controller operation nor increases the controller decision time in real-time operation. Using the same personal computer, the GTO optimization takes 45 mins to converge. It can be concluded that the durations of offline RL training and GTO optimization are in the reasonable range. Knowing that the power system operators can use faster processors and larger memory, the RL training and GTO optimization can be done a lot faster. Therefore, the proposed RL-based PSS can be implemented in real systems without problems.

## Test power systems

The proposed RL PSS is applied to three diverse power systems, namely: SMIB, Kundur’s two-area four-machines, and IEEE 39 bus systems. The test models were built in Simulink-MATLAB using the built-in blocks with proper adjustments.

### SMIB test system

**Fig. 3:**
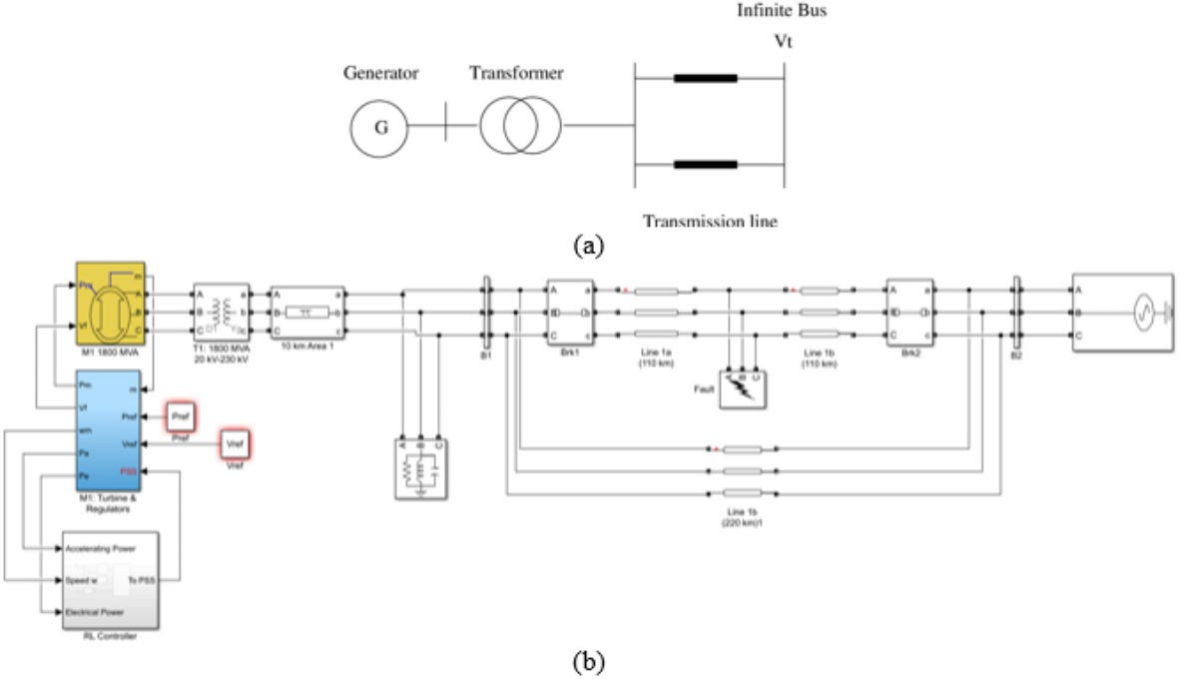
SMIB Model (**a**) single line diagram (**b**) SIMULINK model.

**Fig. 4:**
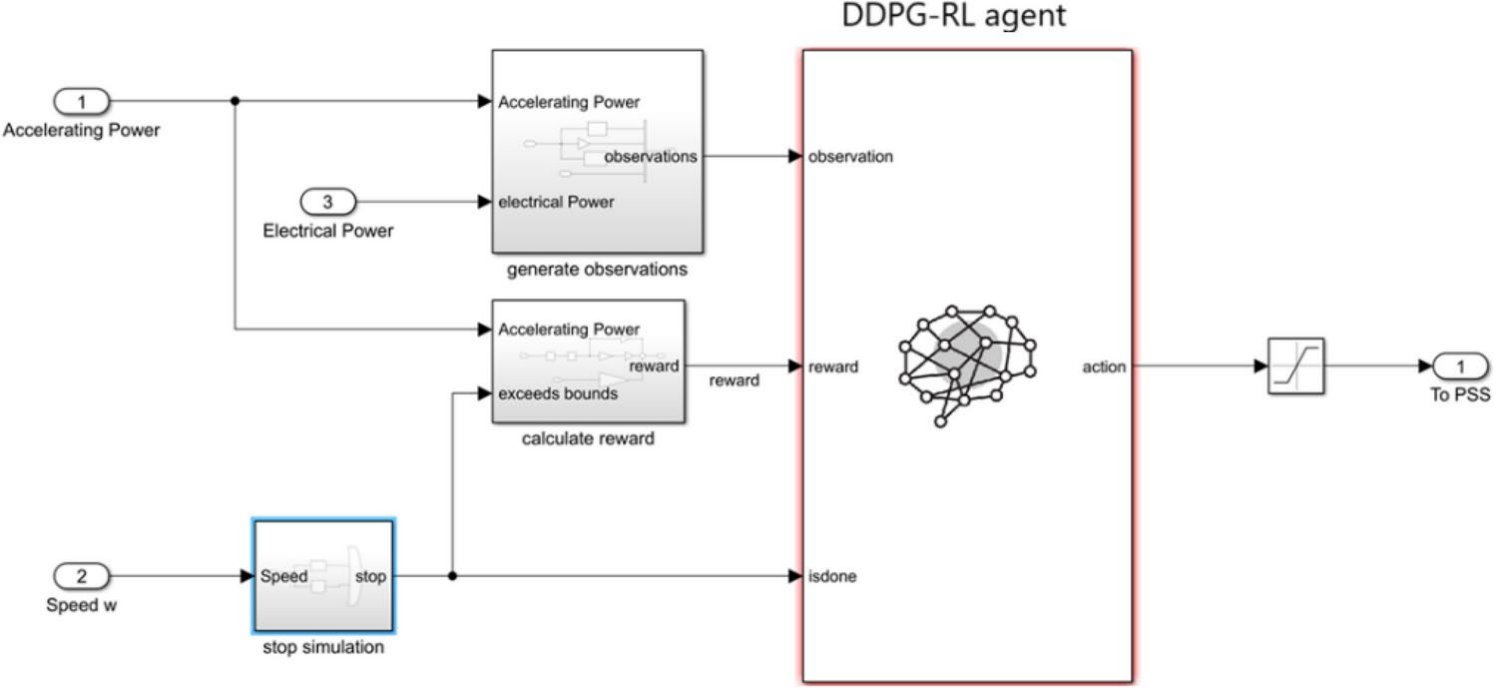
SIMULINK model of RL -PSS for SMIB system.

A 20 kV, 600 MW SMIB is used to test the proposed system. The generator is connected to an infinite bus via a 20 kV/230 kV transformer and two parallel transmission lines. At steady state, the generator produces two-thirds of its rated power (400 MW). This system was chosen to study the case of local rotor angle oscillations. Moreover, the SMIB is equipped with different stabilizers (the proposed and others for comparison). It is also equipped with a none-negative AVR system. Figure 3 shows single line diagram of the SMIB and the corresponding SIMULINK model. Reinforcement Learning Simulink block was attached to the Synchronous machine as a power system stabilizer. The RL Block inputs are the electrical power, weighted accelerating power, and its integral and derivative. The RL Block output is fed to the machines exciter after being Limited to 15% of rated voltage. The RL block (agent) is presented by a deep neural network trained using DDPG algorithm. The SIMLINK model of the proposed RL-PSS is depicted in (Fig. 4).

### Kundur’s two-area four-machine test system

**Fig. 5:**
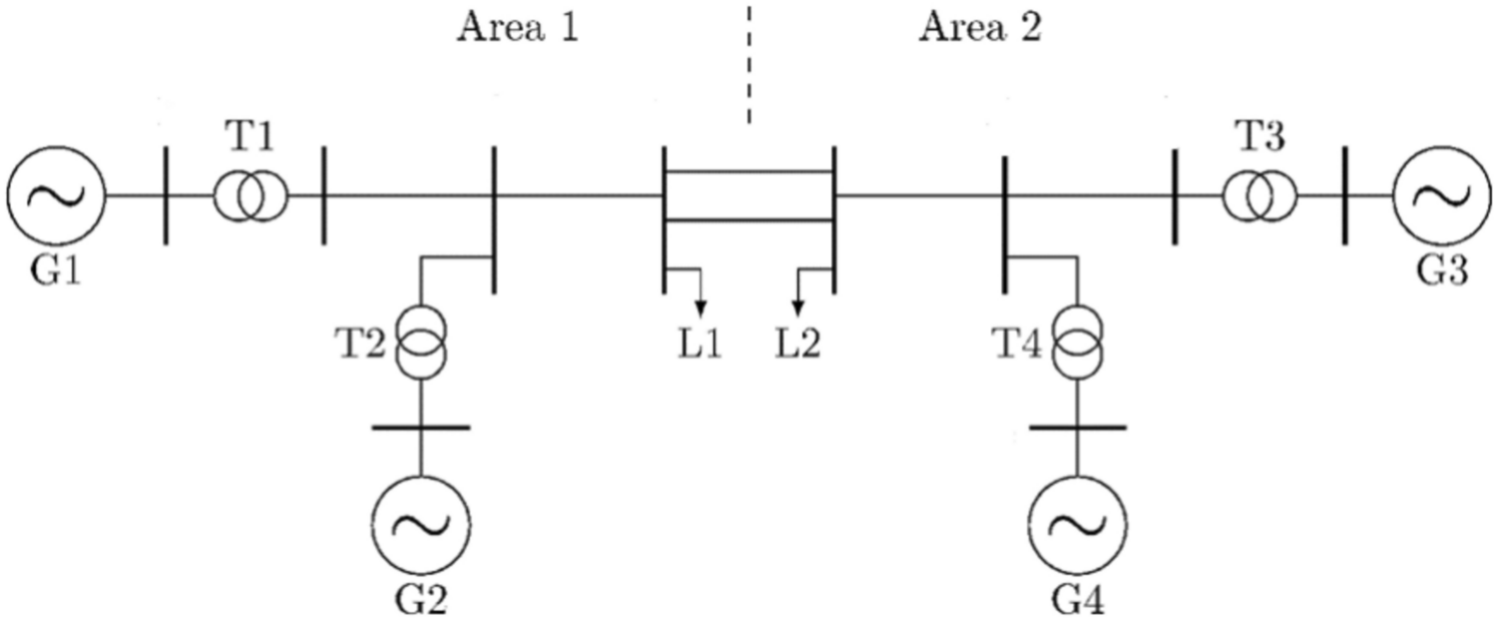
Kundur’s two-area system with four machines.

The proposed stabilizer operation is verified also using the popular Kundur’s two-area, four-machine system. Each area comprises two generators with a capacity of 900 MW for each one^5^. The two areas are connected via double transmission lines, as shown in (Fig. 5). At steady state, the system operates at a stressed condition, such that area 1 transfers 413 MW to area 2. Moreover, all machines are equipped with non-negative AVRs, including the proposed stabilizer and other common power system stabilizers, for comparison. Each of the four machines is equipped with an RL based power system stabilizer as the one described in case study 1. However, the input weighting factors were updated according to the GTO optimizer. This case study is helpful in studying interarea oscillations in a medium size system.

### IEEE 39 bus test system

**Fig. 6:**
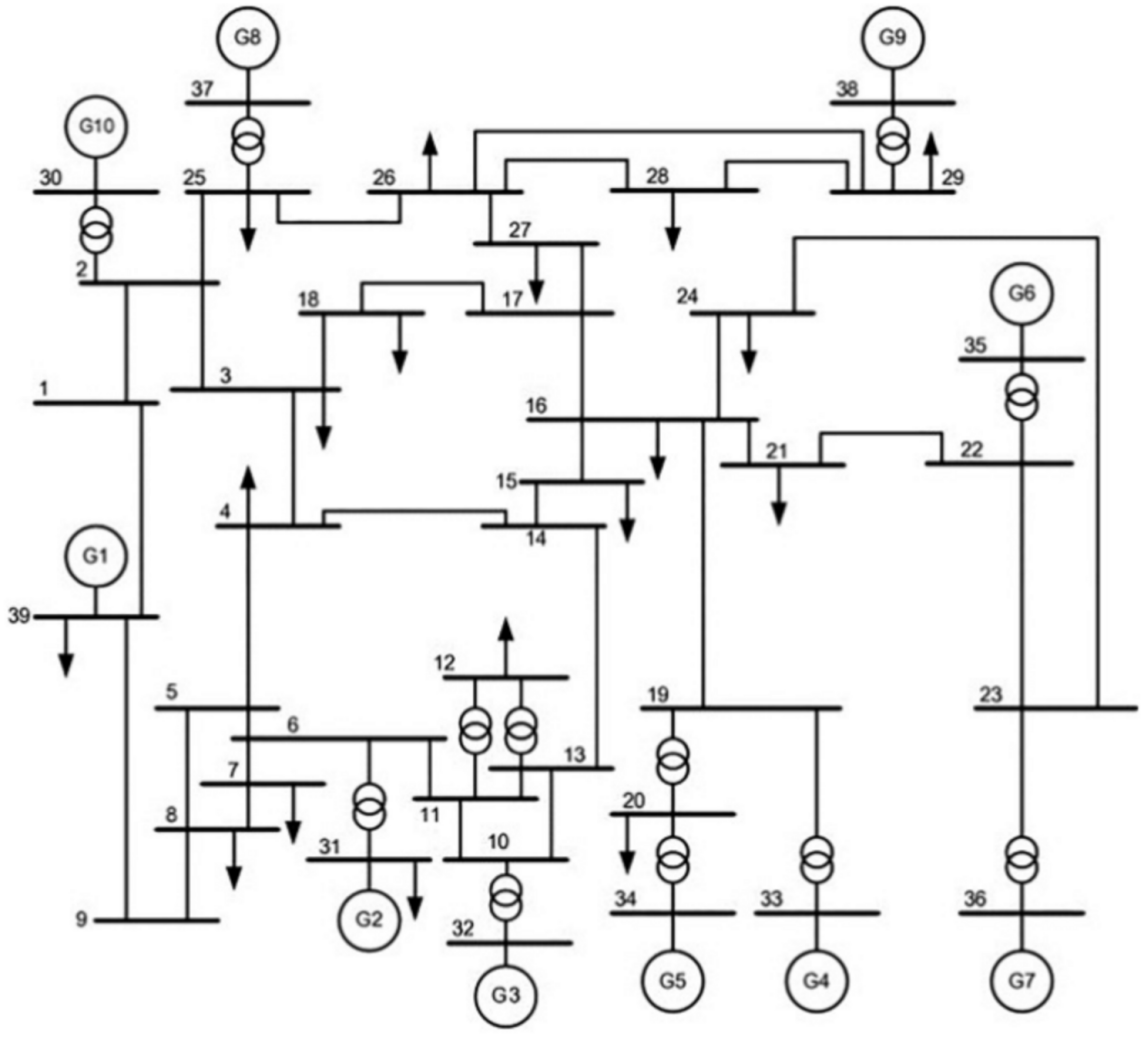
IEEE 39 bus system.

To test the proposed PSS with larger and more complicated systems, the well-known IEEE 39 bus system is utilized. This system consists of 10 generators feeding a total load of 6 GW through a 345 kV network, as depicted in (Fig. 6). The generators of this model differ from the previous two case studies in that their excitation systems and AVRs allow both positive and negative excitation. As case study 2, every generator from the ten is equipped with the proposed RL based PSS like the one described in (Fig. 4). Moreover, all generators are equipped with different types of PSS to compare their performance with the proposed one. The performances of PSSs are compared with respect to damping interarea oscillations in interconnected power systems. Based on a study conducted in^50^ to analyze interarea oscillations in the IEEE 39-system, it was found that a disconnection of line 16–17 due to a 3 phase fault initiates remarkable oscillations. Moreover, multiple generators start swinging off against one another. Such type of faults will be studied in this paper to prove the success of the proposed PSS in damping the oscillations of such problematic disconnections.

## Simulation results

Simulation results for the application of the proposed PSS on the three systems are presented in this section. The proposed PSS’s effectiveness over other PSSs for rotor angle stability is proved using fault simulations.

### Case study 1: SMIB

**Fig. 7:**
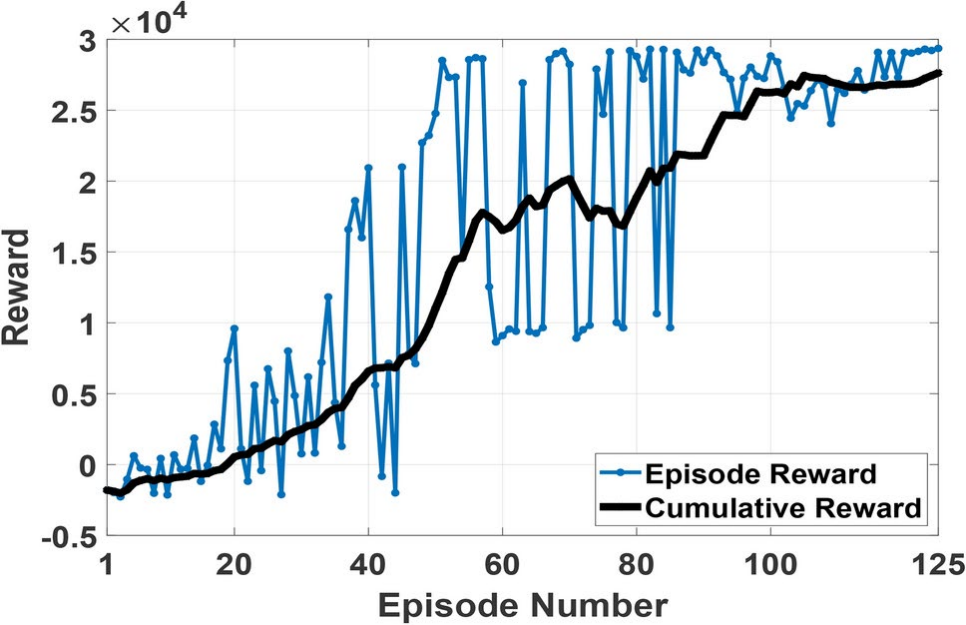
RL agent training process.

**Fig. 8:**
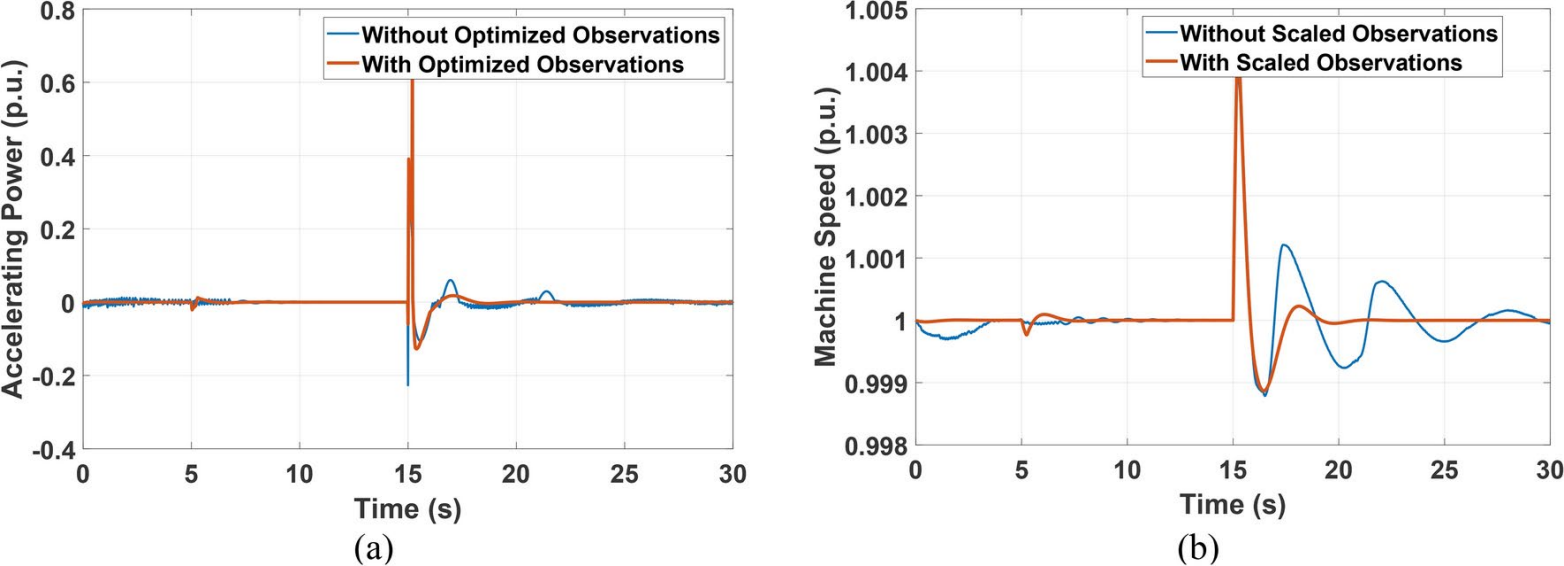
SMIB accelerating power and speed response for pulse increase in voltage reference followed by three-phase fault with and without optimizing the observations scaling factors (**a**) accelerating power (**b**) speed response.

SMIB indicated in Fig. 3 is equipped with non-negative excitation and is used to train the deep neural network-based RL agent. The SMIB system is exposed to a small disturbance (pulse increase in the voltage reference value) followed by a larger disturbance (a three-phase fault) during the same simulation run. This sequence of events ensures the capability of the PSS to operate accurately for different levels of consecutive disturbances. The reward function is a discrete function that rewards each accelerating power sample less than 0.01 with 10 units. Moreover, the controller performance is improved by optimizing the scaling factors of the 4 observation variables. The training process is shown in (Fig. 7), and the optimized observations’ scaling factors using GTO are given in (Table 2). Figure 8 depicts the machine’s accelerating power and speed changes with and without optimizing the observations’ scaling factors. It is clear that optimizing the scaling factors significantly reduces oscillations accompanied by accelerating power and speed changes.

**Table 2:** GTO results for observations’ scaling factors of case study 1.

K1	K2	K3	K4
0.01	3.2351	0	1

#### SMIB system response to 12 cycles of fault disturbance

**Fig. 9:**
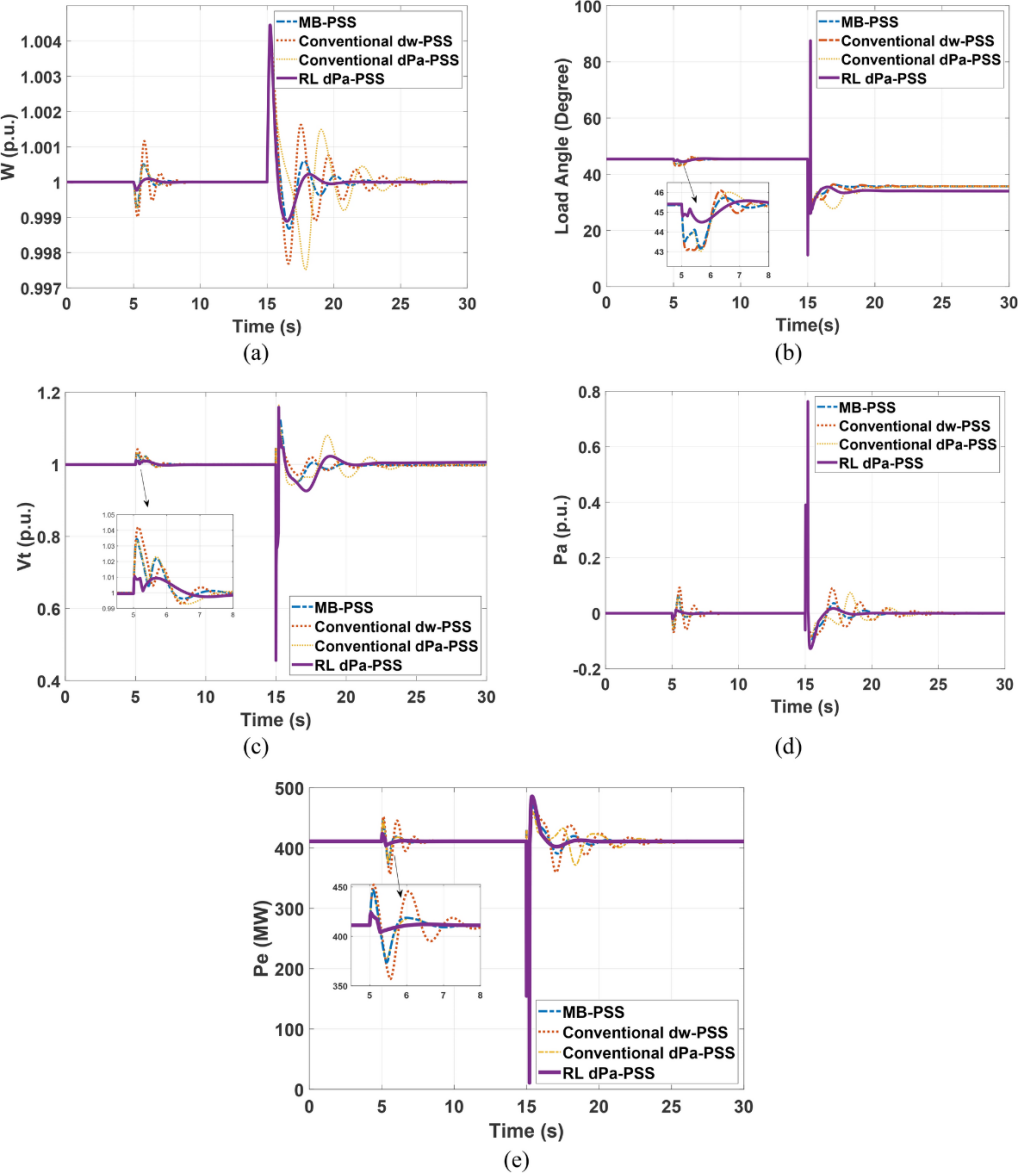
SMIB response under 12 cycles fault using different stabilizers (**a**) speed response (**b**) load angle response (**c**) terminal voltage response (**d**) accelerating power response (**e**) electrical power response.

A small pulse change (5%) of reference voltage is applied to the SMIB system at the second 5. This small disturbance is followed by the disconnection of one line at second 15 due to a 12-cycle three-phase fault. System response simulations show the superiority of the proposed PSS regarding this disturbance, as exemplified in (Fig. 9). The PSS shows better characteristics with respect to damping local oscillations, reducing settling time, and reducing overshoot and undershoot when compared with other common power system stabilizers such as Multiband PSS (MB-PSS), standard dw lead PSS, and the standard dPa lead PSS.

Concerning (Fig. 9), the system shows stability for all 4 controllers. However, the proposed RL-based PSS shows better performance. The maximum Undershoot (MUS), maximum overshoot (MOS), and settling time (Ts) for the four PSSs are summarized in (Tables 3, 4). Table 3 compares the main performance measures of system when subjected to a small disturbance, while Table 4 summarizes the findings when the system is subjected to a 12-cycle 3 phase fault. Table 3 shows better MUS, MOS, and Ts for the proposed RL-based PSS over the other PSSs. Table 4 shows close performance measures for the four PSSs with respect to MUS, and MOS; however, Ts for the proposed PSS is notably smaller, which means that the system reaches full stability in shorter time.

**Table 3:** Comparison between performances of the 4 controllers for SMIB subjected to small disturbance (5% voltage pusle).

SMIB variables	Conventional dw-PSS			Conventional dPa-PSS			MB-PSS			RL-based PSS (DDPG algorithm)		
	MUS	MOS	Ts	MUS	MOS	Ts	MUS	MOS	Ts	MUS	MOS	Ts
W (p.u.)	−0.01	0.0012	4	−0.0004	0.0005	2.6	−0.0004	0.0005	3.25	−0.0002	0.0001	2.5
Vt (p.u.)	−0.008	0.04	4.2	−0.008	0.035	4	−0.005	0.035	4	~ 0	0.01	3
Pa (p.u.)	−0.07	0.09	4.25	−0.006	0.006	2.75	−0.06	0.062	2.75	−0.015	0.01	2.5
Pe (p.u.)	−0.12	0.11	3.5	−0.092	0.09	2.5	−0.092	0.09	2.5	−0.02	0.024	1.5

**Table 4:** Comparison between performances of the 4 controllers for SMIB subjected to large disturbance (12 cycle -3phase short circuit).

SMIB variables	Conventional dw-PSS			Conventional dPa-PSS			MB-PSS			RL-based PSS (DDPG algorithm)		
	MUS	MOS	Ts	MUS	MOS	Ts	MUS	MOS	Ts	MUS	MOS	Ts
W (p.u.)	−0.0023	0.004	15	−0.002	0.004	12	−0.0012	0.004	8	0.001	0.004	4
Vt (p.u.)	−0.043	0.15	7	−0.043	0.15	9	−0.043	0.15	7	−0.5	0.15	6
Pa (p.u.)	−0.09	0.76	10	−0.09	0.76	11	−0.1	0.76	5	−0.12	0.76	3
Pe (p.u.)	−0.12	0.12	9	−0.1	0.12	9	−0.05	0.17	5	−0.024	0.17	3.5

#### SMIB system response to 21 cycles fault disturbance

**Fig. 10:**
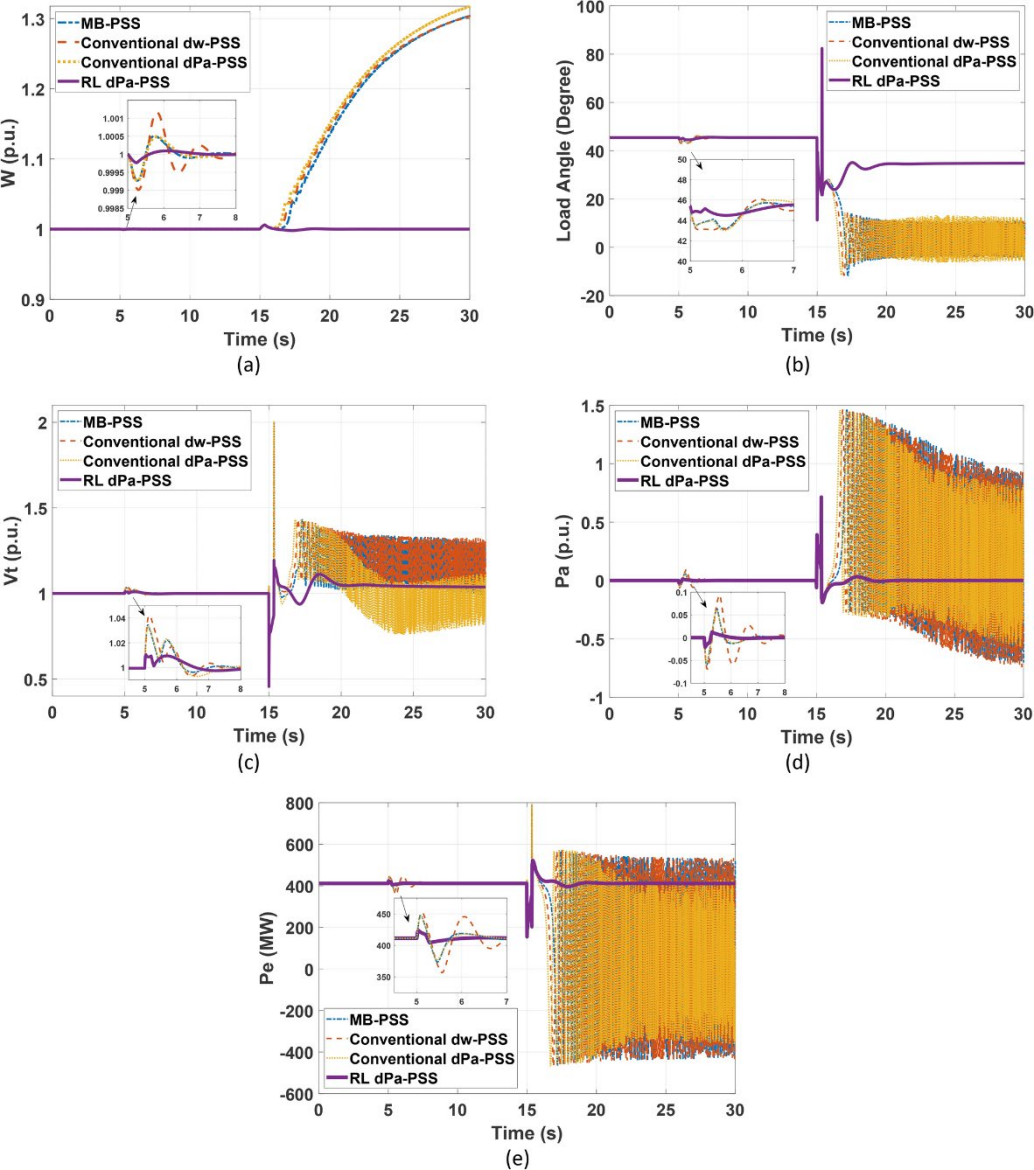
SMIB response under 21 cycles fault using different stabilizers (**a**) speed response (**b**) load angle response (**c**) terminal voltage response (**d**) accelerating power response (**e**) electrical power response.

To test the robustness of the designed PSS under a more challenging fault case, the three-phase fault clearance time is increased to 21 cycles (the critical clearing time for RL case). Figure 10 shows the response of speed, load angle, terminal voltage, accelerating power, and the accelerating power for the SMIB system during the fault. It is clear from Fig. 10 that RL-based PSS continues to stabilize the system even with this problematic fault, while the other PSSs failed to secure system’s stability.

### Case study 2: Kundur’s four-machine system

This system represents an intermediate size. Although Kundur’s system has different characteristics with respect to size, parameters, and dynamics compared with SMIB, the proposed PSS demonstrates good results without the need to retrain the RL agent. You need to only update and fine-tune the scaling factors, hence the GTO is applied again. The new optimal scaling factors are given in (Table 5).

**Table 5:** GTO results for observations’ scaling factors of case study 2.

K1	K2	K3	K4
0.01	4.9786	0	1

**Fig. 11:**
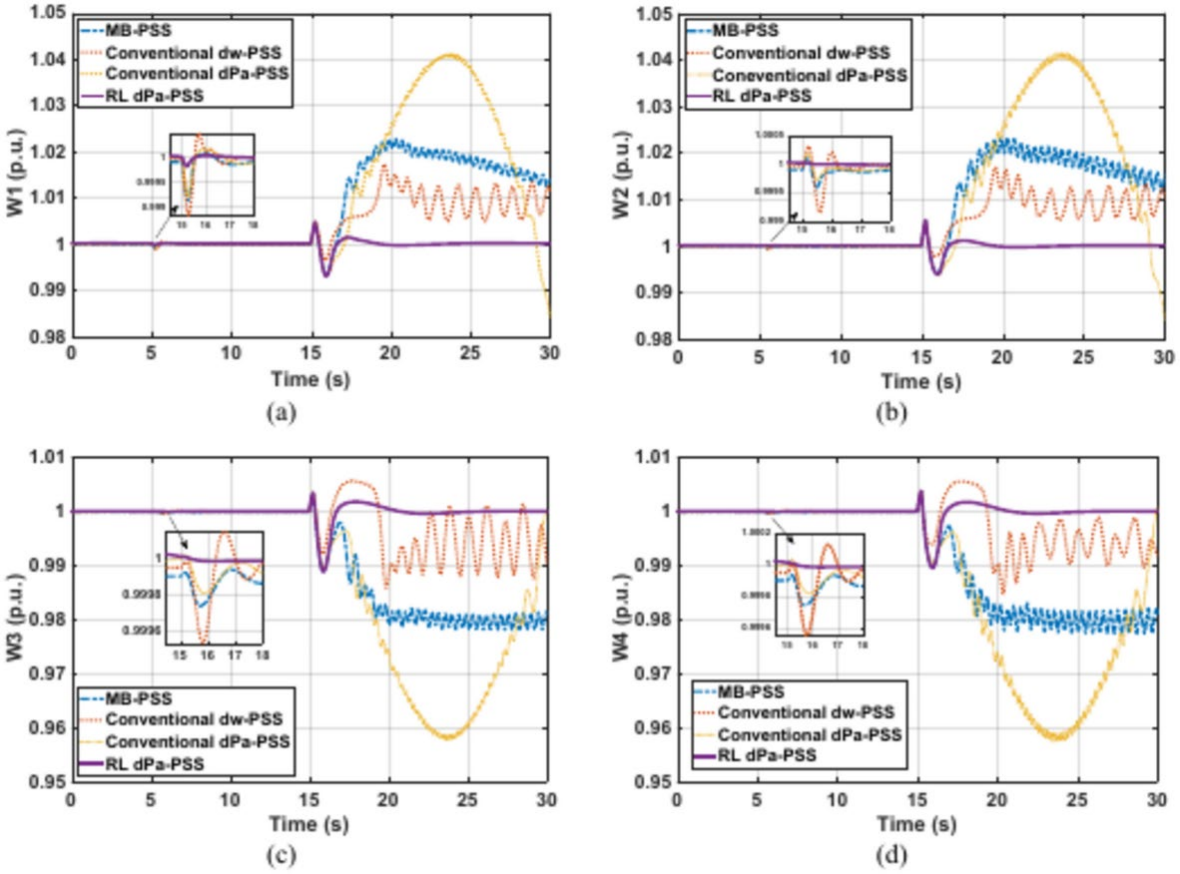
Speed response for Kundur’s system generators when subjected to 15-cycles fault (**a**) generator 1 response (**b**) generator 2 response (**c**) generator 3 response (**d**) generator 4 response.

**Fig. 12:**
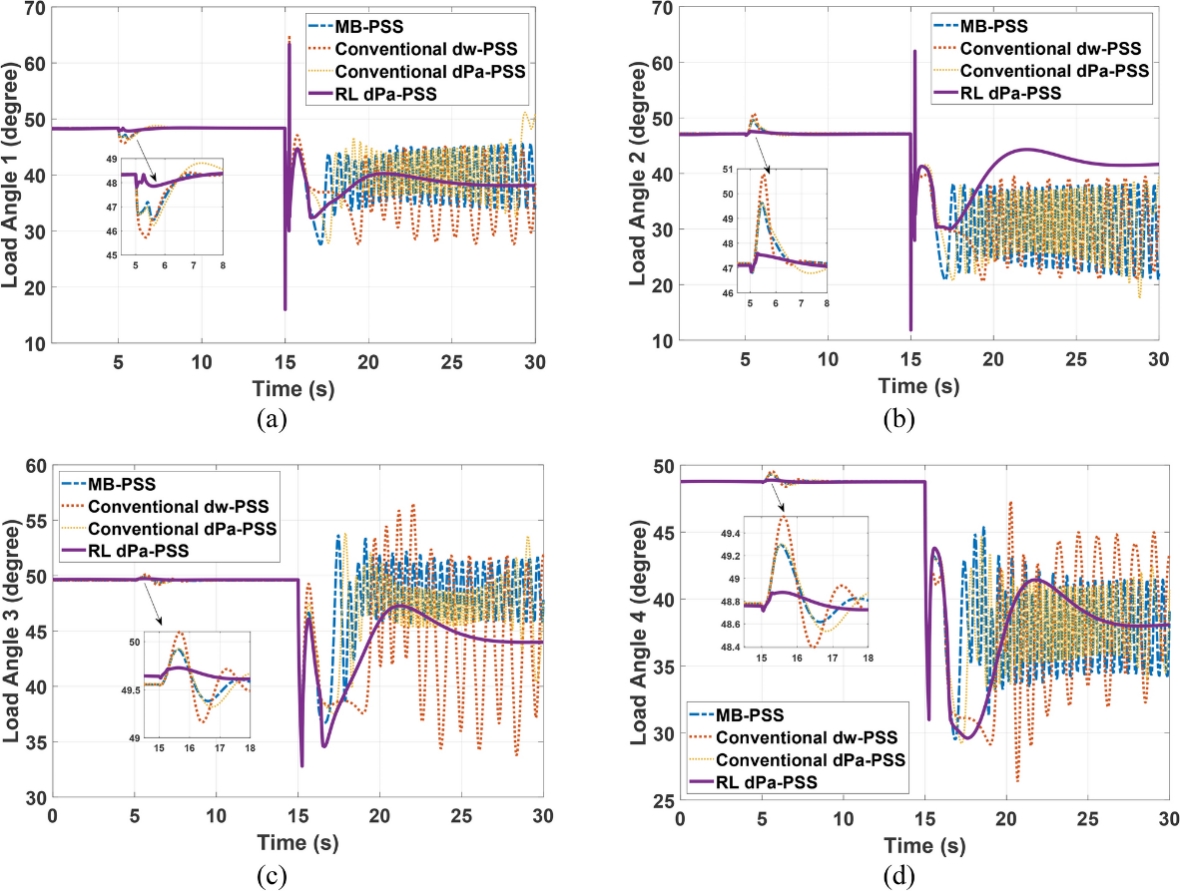
Load angle response for Kundur’s system generators when subjected to 15-cycles fault (**a**) generator 1 response (**b**) generator 2 response (**c**) generator 3 response (**d**) generator 4 response.

**Fig. 13:**
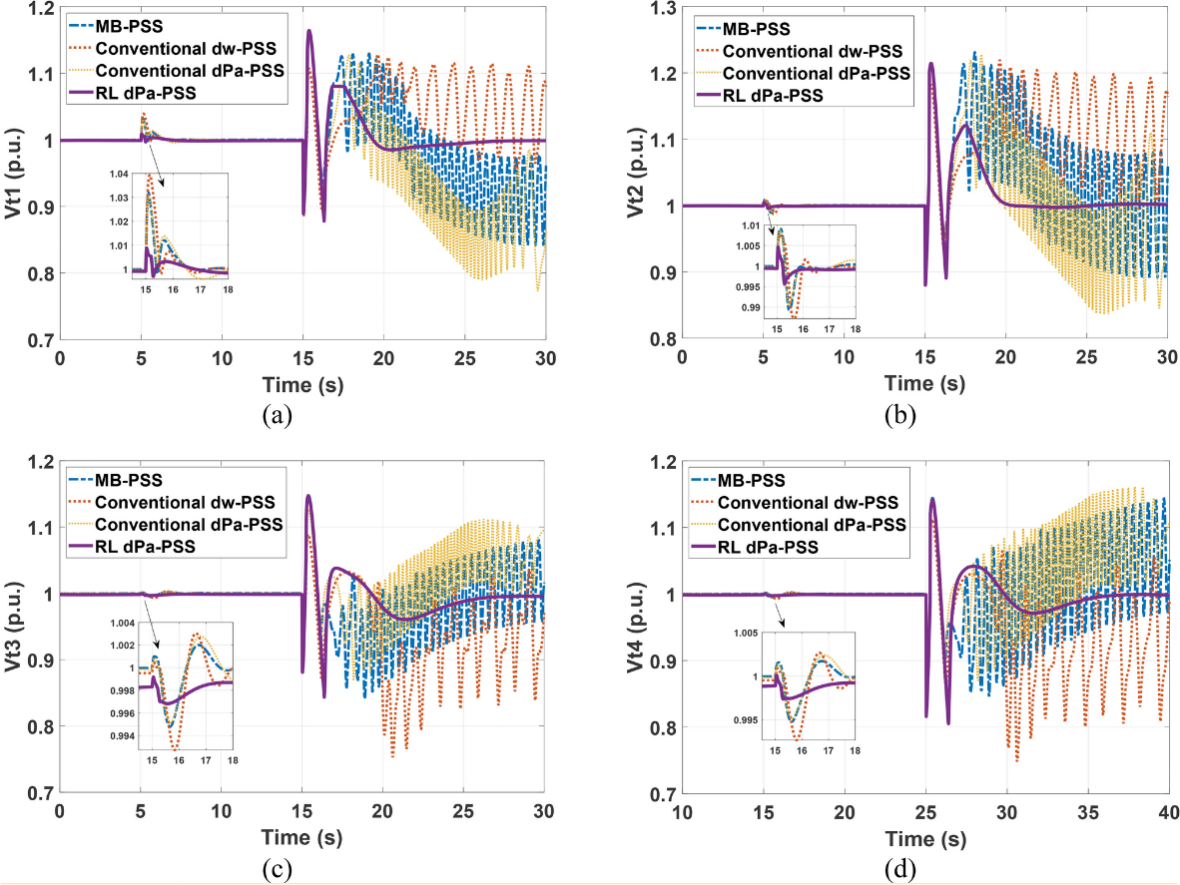
Terminal voltage response for Kundur’s system generators when subjected to 15-cycles fault (**a**) generator 1 response (**b**) generator 2 response (**c**) generator 3 response (**d**) generator 4 response.

**Fig. 14:**
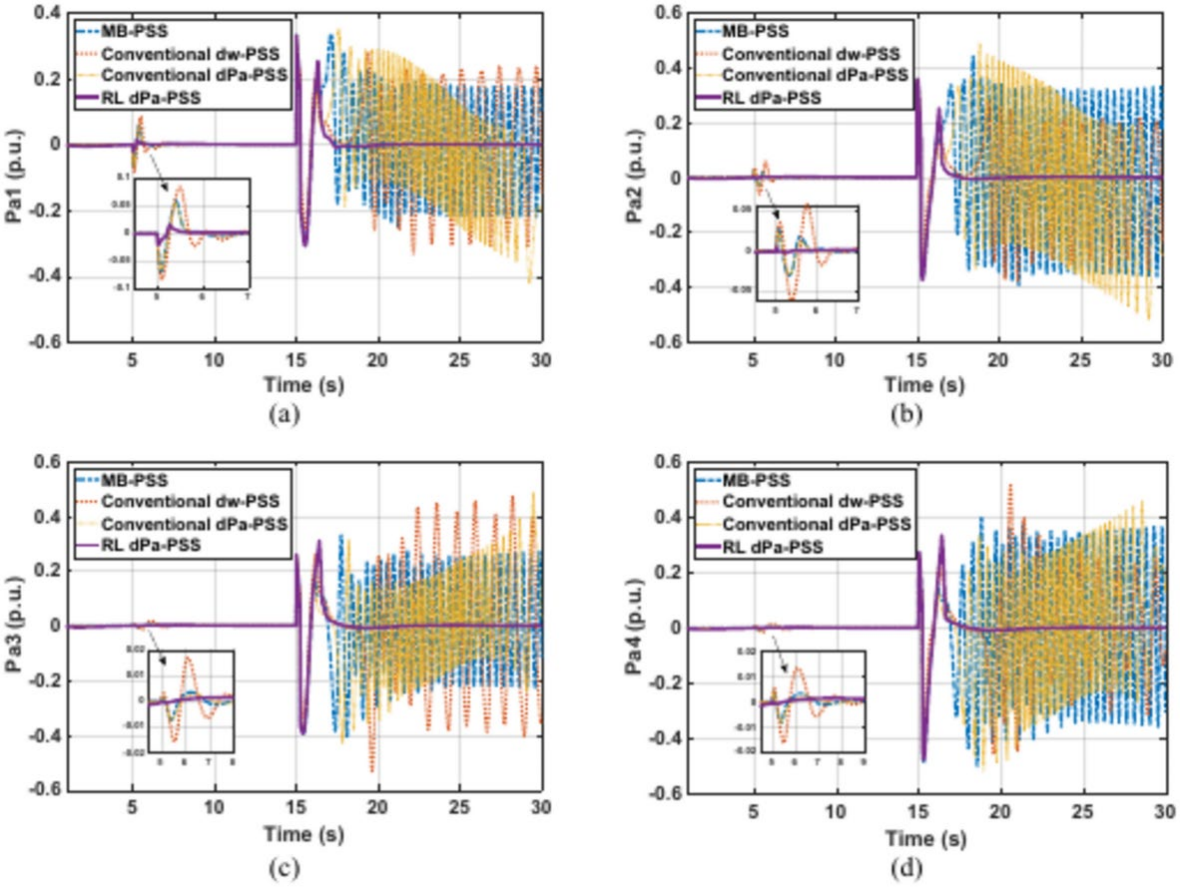
Accelerating power response of Kundur’s system generators when subjected to 15-cycles fault (**a**) generator 1 response (**b**) generator 2 response (**c**) generator 3 response (**d**) generator 4 response.

**Fig. 15:**
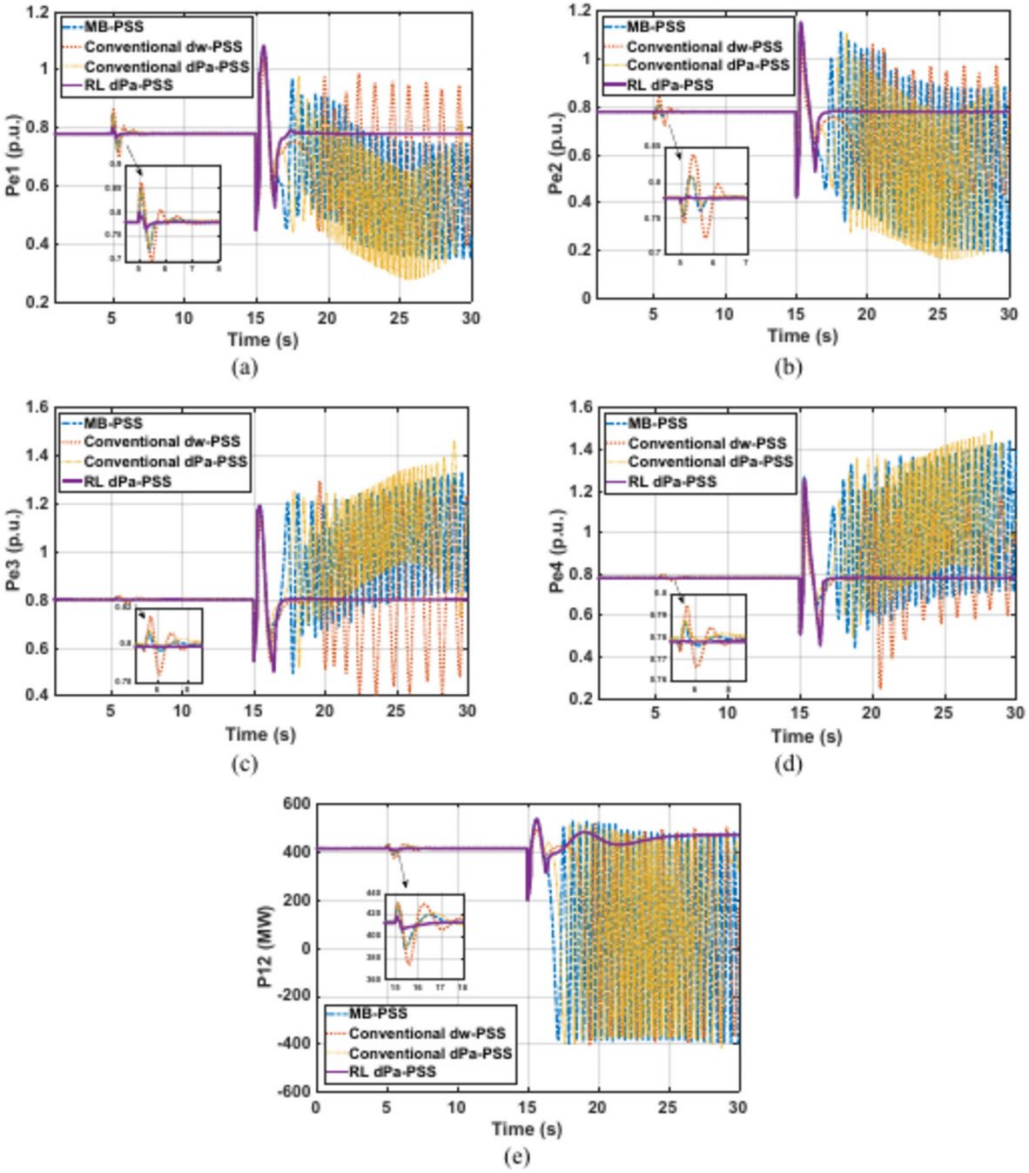
Electrical output powers in Kundur’s system when subjected to 15-cycles fault (**a**) generator 1 power (**b**) generator 2 power (**c**) generator 3 power (**d**) area 1 to area 2 power.

Kundur’s system is subjected to a 5% increase in the reference voltage of G1 at 5 s for a duration of 0.2 s. This disturbance is followed by a three-phase fault at one of the two parallel lines connecting areas 1 and 2 at second 15. The faulty line is removed by the protection system 15 cycles after the fault. Although it was designed for a 12-cycle fault, a 15-cycle fault is considered to show the proposed controller adaptability characteristics and robustness under various fault conditions. Figures 11–14 depict the four generators’ speeds, load angles, terminal voltages, and accelerating powers for different power system stabilizers respectively, while Fig. 15 presents the electrical output powers for all generators and the power transferred from area 1 to area 2. These figures verify that the proposed RL-PSS has the best performance with respect to settling time and undershoot/overshoot for the small disturbance at 5 s. When the system is exposed to a three-phase fault of relatively long period (15 cycles in this case), only the proposed controller can keep the system stable operation when compared with MB-PSS, the dw-PSS, and the dPa-PSS. It exhibits outstanding ability to damp rotor angle oscillations and stabilize the system over traditional PSSs. The power system returns to its reference values promptly with an acceptable overshoot/undershoot and no disastrous oscillations. The compared PSSs couldn’t keep the systems’ stability. Instead, the system suffered from undamped sustained oscillations.

### Case study 3: IEEE 39 bus test system

**Fig. 16:**
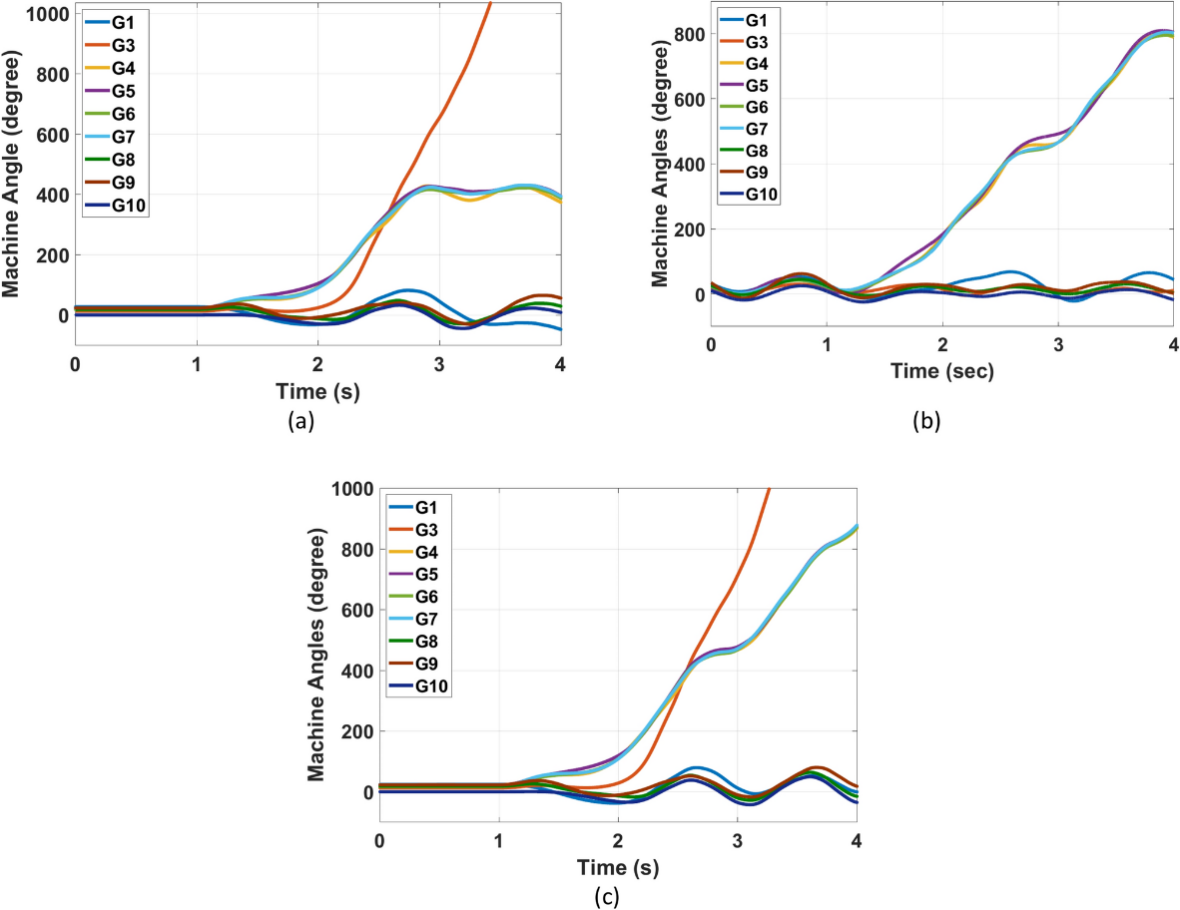
IEEE 39 bus system machines’ angles after disconnection of faulty line 16–17 using common power system stabilizers (**a**) MB-PSS (**b**) Conventional dw-PSS (**c**) Conventional dPa-PSS.

**Fig. 17:**
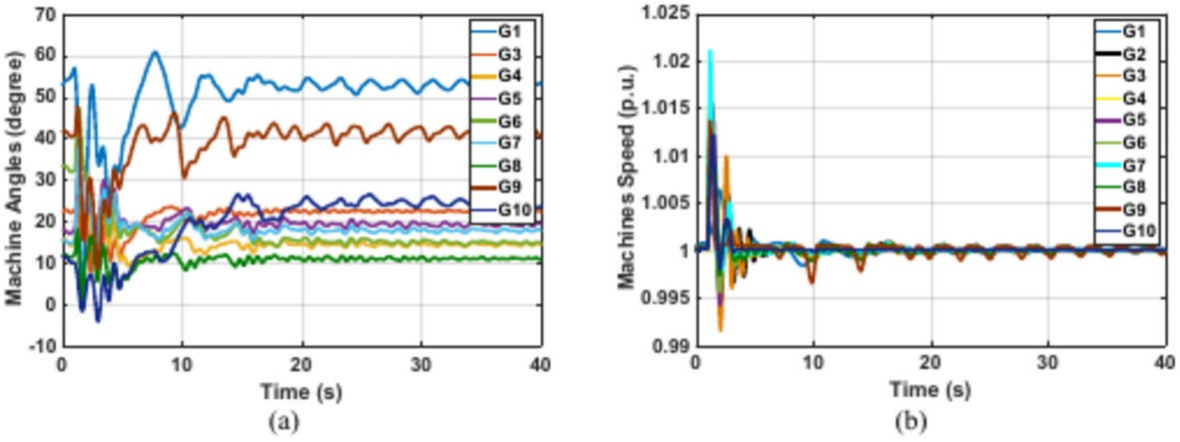
IEEE 39 bus system machines’ angles and speeds after disconnection of faulty line 16–17 using the proposed RL dPa-PSS (**a**) machine angles and (**b**) speeds.

The IEEE 39 bus system represents a highly interconnected power system with multiple generators. Like Kundur’s system analysis, the proposed PSS is able to deal with IEEE 39 bus system disturbances without the need to retrain the RL agent again. Nevertheless, the scaling factors of the PSS input observations are optimized again using GTO. The updated optimal scaling factors for usage in the IEEE 39 bus system are given in (Table 6). Based on a detailed analysis on the IEEE 39 bus system in^42^, problematic faults that initiate oscillations of some generators against each other (i.e., interarea oscillations formation) were identified. The disconnection of line 16 -17 after being subjected to a 12-cycle three-phase fault near bus 16 represents a fault of this problematic nature. This fault is used in this case study to test the RL stabilizer. Time domain simulations of machines’ angles (relative to generator 2) for the different conventional power system stabilizers and the proposed RL PSS are presented in (Figs. 16 and 17), respectively. These Figures prove that the proposed RL dPa-PSS is the only PSS capable of damping the interarea oscillations and stabilizing the system compared with MB-PSS, dw-PSS, and dPa-PSS. The undamped oscillations when utilizing MB-PSS, dw-PSS, and dPa-PSS lead to the division of the machines into groups oscillating against each other. The system gets divided into three groups oscillating against each other for the case of the MB-PSSs and the standard dPa-PSS (G3 alone, G4 G5 G6 G7, and G1 G2 G8 G9 G10), while the system’s machines get divided into only two groups for the case of dw-PSS (G4 G5 G6 G7 and G1 G2 G3 G8 G9 G10). Nonetheless, this division of the machines does not occur in the case of the RL stabilizer.

**Table 6:** Observation scaling factors for case study 3.

K1	K2	K3	K4
0.4485	11.721	0.0024	1

As a summary of the cases studies, the proposed RL-based PSS shows its ability to work effectively on different system sizes with full adaptability and robustness. Although the proposed RL-based PSS was trained using SMIB system, it maintains system stability under different faults within SMIB, Kundur’s, and IEEE 39 bus systems. Nevertheless, the compared PSSs failed to keep stability for SMIB with 21-cycles fault, Kundur’s system with 15-cyles fault, and IEEE 39 bus system with problematic 12-cycles fault. Moreover, the proposed PSS provides good performance characteristics, like over/undershoot and settling time. Table 7 shows a summary for the key parameters for the four cases studies.

**Table 7:** Summary of parameters for the case studies.

Case number	Test system	System size	Disturbance type	Fault duration
1	SMIB system	Small	A small pulse change of reference voltage at second 5 followed by three-phase fault on one line	12 cycles
2	SMIB	Small	Three-phase fault on one line	21 cycles
3	Kundur’s 4-machines system	Medium	5% increase in the reference voltage of followed by a three-phase fault at one of the two parallel lines connecting areas	15 cycles
4	IEEE 39 test system	Larg	Three-phase fault in line 16–17 near bus 16	12 cycles

## Conclusion

A DDPG-RL agent-based PSS is proposed in this research. Scaled values of generator’s accelerating power, a derivative of accelerating power, integration of accelerating power, and generator real power are used as PSS inputs. The PSS performance is improved further by optimizing the observations’ scaling factors using GTO. The proposed stabilizer is tested on SMIB, Kudur’s, and IEEE 39-bus systems. For SMIB equipped with the proposed PSS case, the system response undergoes good overshoot and undershoot with a faster settling time than standard PSSs for different disturbance levels. Moreover, standard power system stabilizers fail to regain system stability after a long disturbance, whereas the proposed PSS succeeds in stabilizing the system after faulty line removal. The proposed PSS shows a superior response to Kundur’s system case study, where it dampens the interarea oscillations and keeps the tie-line power stable under faulty conditions. Nevertheless, the other power system stabilizers failed to keep system stability. Finally, when the proposed PSS was applied to the 10 generators of the IEEE 39 bus systems, it could damp the interarea oscillations and keep the system stability for 12-cycles three-phase faults. Conversely, the system lost its stability with other power system stabilizers for the same fault. The training of the RL agent is done for the SMIB case only; moreover, it does not need retraining for every change in system structure and dynamics. Accordingly, the proposed PSS is adaptable to system changes, which is a salient feature of this stabilizer. Only the observations’ scaling factors are reoptimized for each case study to fully benefit from the excellent PSS adaptability. However, the optimization process is offline and does not affect the PSS operation. Future research includes extending the test systems to include renewable energy sources and deploying the RL-based PSS in real systems.

## Data Availability

Dataset generated during the current study are available from the corresponding author on reasonable request.
